# Pharmaceutical policy Part 1 The challenge to pharmacists to engage in policy development

**DOI:** 10.1186/s40545-015-0027-5

**Published:** 2015-02-10

**Authors:** Norman C Morrow

**Affiliations:** Commonwealth Pharmacists Association, 1 Lambeth High Street, London, SE1 7JN UK

**Keywords:** Pharmaceutical policy, Strategic planning, Medicines management, Pharmacists, Medication safety, Patient involvement, Cost effectiveness, Prescribing, Patient utilisation

## Abstract

Across the world medicines are the ubiquitous technology in the diagnosis, treatment and prevention of disease. Pharmaceutical policy, as part of national health care policy, is concerned with the provision and use of medicines. Pharmacists are critical to the medicines management process, yet are often largely detached from policy development. Logically, they should inform Government policies which impact on their work or where their skills could be best applied to implement health care policy and medicines utilisation in particular. It therefore makes it critically important that the pharmaceutical profession engages with national policy makers and in the strategic planning for health care.

This is the first of two articles directed to this specific issue. Firstly, it identifies a number of the practice challenges for pharmacy and medicines management, their implications for policy and the need for a balanced approach. Drawing from a range of international experiences some key learning points in respect of formulating and implementing national medicines policies are presented. Finally, reference is made to several authoritative evidence bases to inform the development of pharmaceutical practice and medicines management policies.

## Background

In a health care system pharmaceutical policy, as part of national health care policy, is concerned with the provision and use of medicines. It therefore covers medicine development, manufacture, marketing, distribution, pricing and reimbursement, formulary management, pharmacovigilance, patient eligibility, prescribing practice and professional services, particularly pharmaceutical services. Further, regulation policy as it concerns medicines is an integral feature.

Across the world medicines are the ubiquitous technology in the diagnosis, treatment and prevention of disease. The most recent data from The Organisation for Economic Co-operation and Development (OECD) indicates that in some countries medicines account for 20% or more of total expenditure on health [1]. Medicines supply is extensively made through pharmacies or is under pharmaceutical control, not to mention the considerable involvement of pharmacists in medicines management encompassing the entire way that medicines are selected, procured, delivered, prescribed, administered and reviewed to optimise the contribution that medicines make to producing informed and desired outcomes of patient care [2].

Given their pivotal role within this system in providing pharmaceutical services, it is perhaps surprising that pharmacists are often largely detached from government or health service policy development [3], although in some regions, for example, the UK there is a long tradition of Chief Pharmaceutical Officers appointed as professional advisors to Government in England, Scotland, Wales and Northern Ireland.

Pharmacists logically should inform Government policies which impact on their work or where their skills could be best applied to implement health care policy and medicines utilisation in particular. It therefore makes it critically important that the pharmaceutical profession engages with national policy makers and in the strategic planning for health care. That said, engagement with policy makers needs to recognise the possibility of self- interest but also the professional ethic to uphold and assert the patient or public interest.

## Main text

### Common challenges

Across the Commonwealth, despite the diversity in the provisions for health care, with differing levels of development of pharmaceutical services and indeed differing emphases on pharmaceutical practice, a number of common challenges are shared.

Among these are, firstly, the challenge for improved safety in medication use in the light of the substantial body of evidence that details the frequency of adverse medication events, the additional cost to treat, including litigation, their impact on the economy by virtue of working days lost, not to mention the human cost to those who are directly and indirectly involved [4-6]. That is a huge challenge and covers the whole spectrum of therapeutic errors to the risks from products of unacceptable quality, of which counterfeit products cause very substantial threat to the integrity of the medicines distribution chain and the effective treatment of disease. So safety is both a function of the quality of the medication and its appropriate use.

Secondly, there is the challenge of greater access to medicines, affordability and improved cost effectiveness in their use, particularly in the context of managing communicable diseases and managing non-communicable chronic disease states [7]. International comparisons clearly demonstrate there are considerable differences in the expenditure on medicines, proportional to GDP, as there are differences in the range of medications available, particularly newer high tech medications [8]. But there can also be huge differences within countries allied to the profiles of prescribing practice [9,10]. This raises a major question as to how to tackle the problem of ensuring consistent quality prescribing that is evidence-based and delivers the desired outcomes in individual patients at an affordable cost?- the right medicine to the right patient at the right dose/time and at the right cost.

It might, therefore, be argued that the key objectives are access to cost-effective medicines and excellence in prescribing practice coupled with the condition of being protected from danger, risk or injury.

Thirdly, and linked to the former, is the challenge of responding to and encouraging patient involvement in decision making about their treatment and adherence to it, in order to achieve optimal healthcare outcomes [11,12]. Do our policies encourage a model of cooperative health care that seeks to achieve active involvement by patients, professionals, caregivers, and others across the continuum of care on all issues related to an individual’s health? Arguably, such an approach holds promise to improve outcomes, reduce errors, increase patient satisfaction and improve the cost of care. One excellent example is **DOTS** (directly observed treatment, short-course), the WHO recommended tuberculosis control strategy [13]. Further, the term ‘e-patients’ has been coined to describe individuals who are **e**quipped, **e**nabled, **e**mpowered and **e**ngaged in their health and health care decisions [14]. So it is necessary to think in terms of equal partnership between ‘e-patients’ and health professionals and systems that support them.

Fourthly, particularly in the context of primary care pharmacy, is the challenge of developing appropriate remuneration systems. Where should the focus be – paying for the product, paying for the service or paying for the performance? Aspiring to a quality- based system means that performance will be critical with measurable outcomes linked to services. Arguably, this has been furthest advanced through the successive national agreements between the Australian Government and community pharmacists [15].

In effect, policies need to put much more emphasis on outcomes than processes and in the context of pharmaceutical provision identifying those quality indicators and measures that will demonstrate impact [16].

### A balanced perspective

In the context of these challenges, it will be important to ensure and maintain a balanced perspective or approach. If, for example, pharmacists keep the patient or person at the centre of their thinking they will be less liable to become self-serving in what they do or offer. Evidence of this patient focused approach is illustrated with reference to the new strategy in Northern Ireland, ‘Making it better through pharmacy in the community’ [17] (Table 1).

**Table 1:** **Strategic pharmaceutical goals in patient-focused care** [17]

**Theme**	**Strategic goal**
Helping people to gain better outcomes from medicines	To ensure that throughout life, in accordance with their clinical needs, people have access to timely, safe, quality assured medicines supplied with appropriate advice and support to help them gain the best outcomes from their treatment and avoid harm.
Helping people to live longer, healthier lives	To provide people with access to advice and support from pharmacists in the community promoting, public health, self care, improved health and wellbeing and preventing illness.
Helping people to safely avail of care closer to home	To provide improved access to clinical expertise and interventions for patients closer to home by making the best use of the skills of pharmacists working together with/alongside other healthcare professionals in the community.
Helping people to benefit from advances in treatment and technology	To support better health outcomes for patients through advances in medicines treatments and technology.

Cost effectiveness, as mentioned earlier, is critically important and high on health care providers’ agendas. Currently, much of health care thinking and planning is finance dominated. Indeed to take a short term ‘balancing the books ‘approach can easily stifle innovation and development as well as more radical policy making. This comment by the President of the Slovak Republic in an address to the National Council of the Slovak Republic in June 2005 makes the point:

‘Our citizens expect the health care reform to introduce more human dignity, equality, solidarity, but also better professional quality, ethics and care. However, the reality is often totally different. The reform is presented to the people as a purely economic problem, a problem of lack of money, whereby there is almost no mention of efforts aimed at improving the health of the people…….Health is the ultimate value. We must not reduce it to a market commodity’ [18].

Thirdly, in a world increasingly dominated by technology with there is enormous ability and capacity to collect, analyse and share information; also an ability to communicate almost instantaneously across the world. Such technology offers a new dimension for interaction between patients and professionals that could advantage the management of their health and social care. It is a tool, not an end in itself, and policy needs to address the utility of information technology to support medicines management, particularly to support patient adherence with medication [19,20].

Finally, in seeking to achieve more responsible use of medicines collaboration is needed, not simply the engagement with patients mentioned earlier, but collaboration between the health professionals themselves, particularly in the medicines arena where there is clear multidisciplinary involvement. Pharmacists should take the lead responsibility across the whole domain of medicines management but that is not an exclusive role and it will be important to build, encourage and develop a team based approach where there is mutual respect for the expertise that each brings with corresponding due responsibility [21,22]. So it will be important to consider policy that promotes integrated care based on collaborative team working and that ensures that there is sharing of records and pursuit of joint models of remuneration rather than siloed approaches.

### National medicines policies

Hoebert and her colleagues have reported on the evolution and development processes concerned with the formulation and implementation of national medicines policies (NDP), illustrated with reference to Sri Lanka, Australia, the Former Yugoslav Republic of Macedonia and South Africa [23].

Their work evidences some important learning points in respect of formulating and implementing such policies.

1. **The development of a NDP is complex and country specific.** Complexity is a product of financial considerations, changing patterns of morbidity, public and private sector interests, legislation, the presence or absence of health insurance schemes, health care reform and the globalisation of the pharmaceutical market. So policy formulation must take account of those factors existing within a specific (country) con**t**ext and those that impinge upon it from outside. While national NDPs invariably share a number common objectives e.g. access, quality, effectiveness and affordability, there are also individual differences e.g. the establishment or maintenance of a strong local manufacturing base.
2. **The development of a NDP often requires a critical political window or trigger to initiate the process.** It may be the coalescence of a number of stakeholders eg. patients, NGOs, regulators, health care professionals etc. arguing in favour of a NDP. Alternatively, it may be the ambition to enable universal access to medicines or equity of access in divided societies, or in response to a critical political or safety event or simply to seek to bring coherence or governance to a fragmented situation. Pharmaceutical policy formulation can therefore take advantage of the prevailing circumstances to seek to offer better care for patients. Moreover, the process of development of a NDP is as important as the final policy document in ensuring collective ownership, broad stakeholder engagement being a critical element to success.
3. **The development of a NDP must be accompanied by a clear implementation process with monitoring.** Many policies fail because there is no planned and monitored implementation process. A new policy doesn’t implement itself, it needs resources, drive and determination to seed it in place. It also requires the development of milestones, indicators and targets which will mark and measure its outworking. This will allow for policy evaluation and the identification of future steps. Hoebert et al. [23] put it in these terms:- ‘Unless there is a performance related budget linked to the policy, adequate implementation (and monitoring) is unlikely to occur.’

Some NDPs are also more explicit in their links to pharmaceutical services as vehicles through which the policy is to be effected. For example, the recent Medicines Policy 2020 in Finland set out its policy objectives in these terms:

- Pharmaceutical service is a part of the social welfare and healthcare service system.
- Pharmaceutical service is of high quality, efficient and cost- effective.
- Rational pharmacotherapy and good medication safety enhance the wellbeing of the population, improve public health and decrease the healthcare expenditures.
- Pharmaceutical research enhances health, wellbeing and employment.
- Veterinary pharmaceutical service safeguards public health and promotes the wellbeing of people and animals [24].

### Evidenced-based policy

In October 2012, the Ministry of Health of the Netherlands convened a Ministerial ‘Summit’ that brought together Health Ministers and other senior policy makers from a range of countries around the world to discuss the theme of ‘The Benefits of Responsible Use of Medicines – Setting policies for better and cost-effective healthcare’ [25]. The Summit focused on:

- Responsible use of medicines (the right medicine in a correct dose for the right patient at the right time)
- Unlocking the full potential of prescribers, pharmacists, nurses and other healthcare professionals
- Sharing and translation of best practices and experiences from countries with measurable successes

The Summit was informed by two reports commissioned by the Dutch Ministry of Health - WHO: ‘The Pursuit of Responsible Use of Medicines: Sharing and Learning from Country Experiences’ [26] and the IMS Institute for Healthcare Informatics: ‘Advancing the Responsible Use of Medicines – Applying Levers for Change’ [27]. In addition, The European Directorate for the Quality of Medicines and Health Care (EDQM) prepared a report on its work regarding the quality philosophy and working method of pharmaceutical care entitled ‘Pharmaceutical Care - Policies and Practices for a Safer, More Responsible and Cost-Effective Health System’ [28].

These provide a useful and important evidence base for the formulation of pharmacy and medicines policy, supported by a Report from the Summit itself [29]. This has been recently followed up in August 2013 by the Irish Department of Health and Children who convened a symposium attracting over 80 senior officials from international organisations and government Ministries worldwide with responsibility for pharmacy and pharmaceutical policy and regulation. They met to address the issue of ‘Achieving Responsible Use of Medicines – Real Patients, Real Policy, What Really Works?’, focusing on practical solutions and sharing best practice [30].

In the southern hemisphere similar discussions have taken place, focusing on the wide range of issues concerned with the development, implementation and evaluation of national medicines policies [31].

What is clear from these publications and the Summit and Symposium discussions is that initiatives to promote the responsible use of medicines have real potential to improve outcomes for individual patients. The responsible, cost-effective use of medicines also provides the capacity for health systems to fund existing services and invest in new innovative medicines. While much emphasis in the past has been on prescribing and procurement i.e. around healthcare professional responsibilities, much more attention needs to be paid to patient utilisation of medicines, indeed their place in the decision making aspects of their treatment - ‘no decision about me, without me’. Allied to this is the need for a more integrated approach to care with the different healthcare professionals collectively applying their skills to maximise the benefit to the patient.

Clearly there is opportunity for greater pharmaceutical involvement to benefit health care and indeed acknowledgement of the skills that pharmacists have that could be more effectively deployed. Indeed there is evidence of Government recognition of that potential. For example, in the UK ‘pharmacy is now seen as a new centrally important bridge between the clinician and the patient....... The government wants to see more pharmacist-led clinical services, including pharmacists managing chronic diseases, and greater involvement of pharmacists in promoting the safe and effective use of medicines, healthy living and health literacy. The future is bright for pharmacists. The greater clinical status pharmacists will achieve should improve both access to, and the quality of, medicines use in the UK’ [32].

## Conclusion

Pharmacists need to address the challenge of healthcare policy making, bringing their knowledge and experience to inform the direction and strategic delivery of services. Of critical importance is the application of the evidence base to inform policy, including the experience of policy making in nation states. This will also allow the articulation of specific application of pharmacist skills and services towards the fulfilment of policy objectives. It is, of course, a two-way street that in charting direction comes the challenge of demonstrating effectiveness.
